# Crosswalks among stewardship maturity assessment approaches promoting trustworthy FAIR data and repositories

**DOI:** 10.1038/s41597-022-01683-x

**Published:** 2022-09-21

**Authors:** Ge Peng, Wendy S. Gross, Rorie Edmunds

**Affiliations:** 1 Earth System Science Center/Interagency Implementation and Advanced Concepts Team (IMPACT) Project of NASA’s Marshall Space Flight Center (MSFC), The University of Alabama in Huntsville, Huntsville, AL USA; 2 Previously NOAA’s Cooperative Institute for Satellite Earth System Studies (CISESS), North Carolina State University, at NOAA’s National Centers for Environmental Information (NCEI), Asheville, NC USA; 3 NOAA’S National Centers for Environmental Information (NCEI), Boulder, CO USA; 4 DataCite, Welfengarten 1B, 30167 Hannover, Germany

**Keywords:** Environmental sciences, Research management, Biological physics, Hydrology, Data publication and archiving

## Abstract

Various maturity assessment approaches have been developed to help research data repositories effectively manage their holdings at both the organizational and dataset levels. Repositories can use these approaches as self-assessment tools—potentially leading to formal certification—to benchmark the maturity of their data holdings, highlight gaps in their practices, and improve their sustainability. Understanding the differences among these assessment approaches can provide beneficial information on stewardship best practices for supporting FAIR data managed by Trustworthy Data Repositories. However, it is a daunting task due to diversity in the perspectives of the approaches and the potential for subjective interpretation of individual criteria. In this article, we outline the commonalities and distinctions of three established assessment approaches: i) CoreTrustSeal Trustworthy Data Repositories Requirements, ii) Data Stewardship Maturity Matrix, and iii) FAIR Guiding Principles. Strong correlations are found in data discovery, accessibility, interoperability, and usability due to overlapping requirements in digital object management. The study also reveals that the complexity of the approaches can lead to a large variety of inferred crosswalks among them.

## Introduction

Scientific data repositories are increasingly facing requirements to ensure their digital data holdings are findable, accessible, interoperable, and reusable (i.e., FAIR), and to show their trustworthiness in managing and preserving these data holdings for the long term—namely, that they are a Trustworthy Data Repository (TDR). To sustain and enhance best practices for data management and governance, and to meet the ever-evolving needs of user communities, data repositories need the ability to evaluate stewardship maturity from the organizational level down to individual datasets.

The trustworthiness of individual repositories has been the topic of study for the data management and preservation community for many years, with various maturity assessment approaches developed to help effectively steward data holdings. These approaches identify gaps in a repository’s digital object management and technological processes. By using them as self-assessment tools—potentially leading to formal certification—data stewards and dataset providers can obtain a benchmark on the current state of accessibility and usability of the data holdings, highlight areas that necessitate further investment, and develop pathways to raise stewardship maturity^1^. Ultimately, a data repository can improve its sustainability to reliably serve its stakeholders into the future.

With multiple maturity assessments to choose from, understanding the commonalities and differences among them is a daunting task as they may vary greatly in terms of quality perspectives and attributes^2,3^. This article details the synergies and distinctions of the three data management and stewardship maturity assessment approaches listed below, in terms of crosswalks among them, leveraging off overlaps and finding areas of collaboration.

Disciplinary-focused repositories need to be able to draw from maturity models that examine trustworthiness at different scales, from the dataset to repository-wide level, and both now and long term. In addition, the models should themselves be trustworthy in providing an accurate and useful picture of a repository’s status, and be well-respected by its designated community and peers. The authors choose the below models as meeting this challenge. These three different approaches have been well-established and shown to help data stewards and organizations improve their organizational capability and practices for long-term preservation and access of their data holdings. Knowledge of the synergies among them is valuable to scientific data repositories and the wider data stewardship community.

### CoreTrustSeal Trustworthy Data Repositories Requirements (CTDRR)

**Fig. 1:**
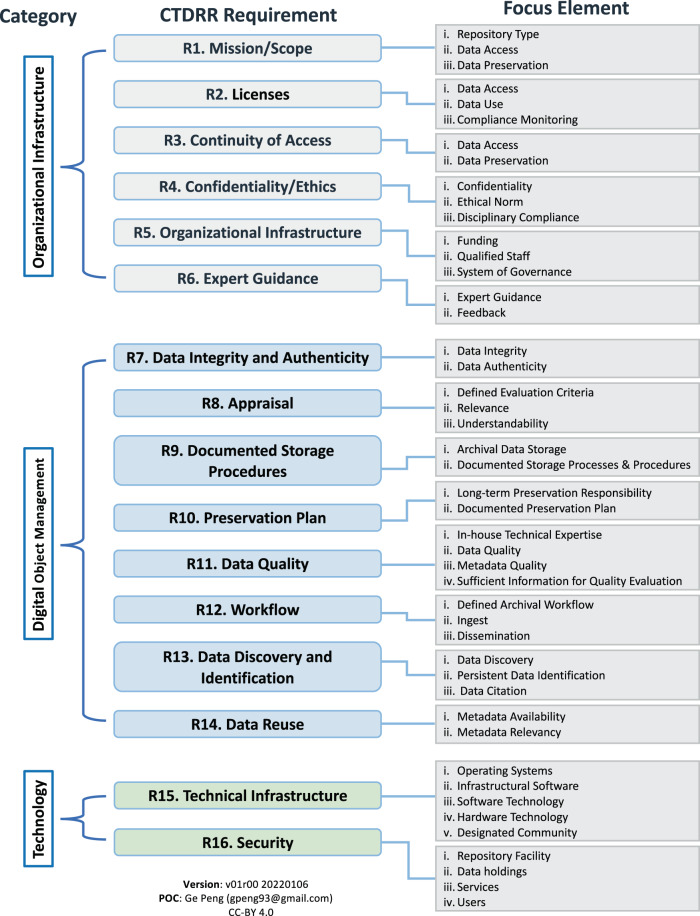
Diagram showing focus elements associated with the CoreTrustSeal Trustworthy Data Repositories Requirements (CTDRR). As noted in the diagram, the sixteen CTDRR requirements are grouped into three categories: Organizational Infrastructure (R1-R6), Digital Object Management (R7-R14), and Technology (R15-R16).

The most well-established evaluation method is ISO 16363^4^, which is based on the Open Archival Information System (OAIS) Reference Model, and establishes comprehensive audit metrics for what a repository must do to be certified as a TDR (see also^5^). However, while it is desirable to be ISO 16363 certified, the time and financial burden tends to keep many organizations from pursuing it. The World Data System of the International Science Council, in collaboration with the Data Seal of Approval and the Research Data Alliance, thus developed a set of 16 core CoreTrustSeal Trustworthy Data Repositories Requirements (CTDRR) for certification of repositories at the core level as a solid step towards meeting the ISO 16363 standard^6–8^. The focus elements for each requirement are shown in Fig. 1, based on the latest version described in CoreTrustSeal (2019)^8^. Currently, 183 institutional or disciplinary repositories have been accredited as core certified repositories (Latest statistics can be found at: https://www.coretrustseal.org/why-certification/certified-repositories/).

Although existing prior to their creation, both CTDRR and ISO 16363 are embodiments of the Transparency, Responsibility, User Focus, Sustainability, and Technology (TRUST) Principles^9^. The TRUST Principles are formulated in a high-level way that brings the concepts around repository trustworthiness under one overarching umbrella. In this regard, they have been designed as a communication tool on the important roles of repositories, rather than offering comprehensive and specific measurements, to ensure long-term stewardship of research data. Therefore, the authors believe that it would not be appropriate to include a crosswalk between the TRUST Principles and any of the chosen assessment approaches.

### Data Stewardship Maturity Matrix (DSMM)

**Fig. 2:**
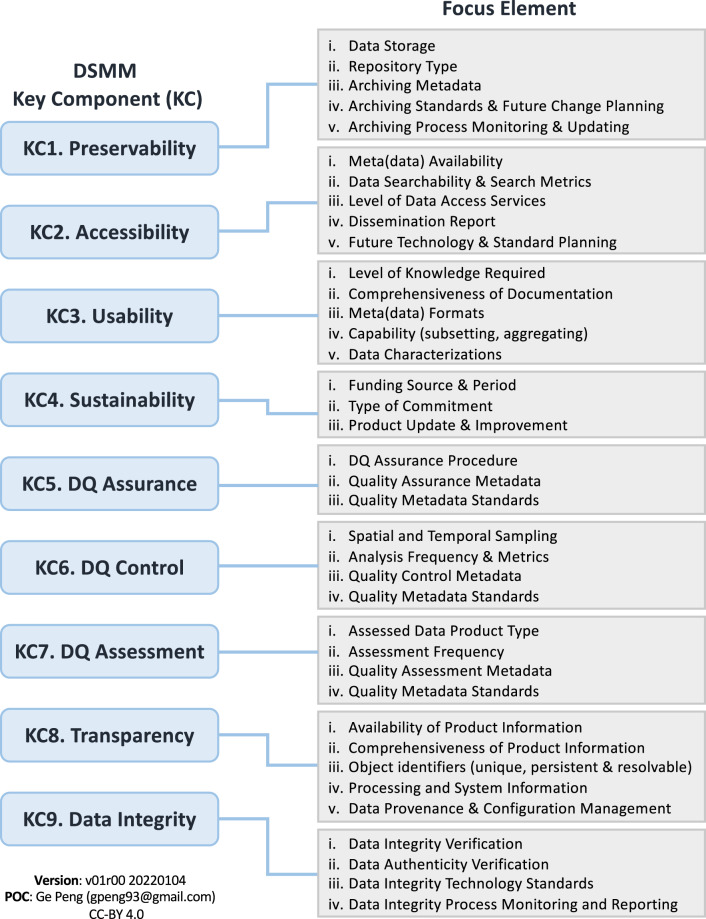
Diagram showing focus elements associated with the NCEI/CICS-NC Data Stewardship Maturity Matrix (DSMM) Key Components (KCs).

Similar to CTDRR, the scientific data stewardship maturity matrix (DSMM^1^) is within the scope of the OAIS Reference Model, but it is instead focused on the individual dataset level. DSMM was developed jointly by the National Centers for Environmental Information (NCEI), the official archive of the U.S. National Oceanic and Atmospheric Administration (NOAA), and the Cooperative Institute for Climate and Satellites–North Carolina. NCEI is an authoritative source for environmental data and information, including climate data records that have been utilized in national and international climate monitoring and assessment activities. DSMM assesses the maturity of stewardship practices in nine Key Components (KCs) to help ensure that individual digital datasets are of assured scientific quality; adequately preserved and documented; and that they remain accessible, usable, and current to end users. The focus elements covered under each DSMM KC are shown in Fig. 2. So far, DSMM has been adopted or adapted by several Earth Science organizations^10,11^. In particular, the Stewardship Maturity Matrix for Climate Data (SMM-CD) is now one of the three building blocks of the World Meteorological Organization (WMO)’s high-quality framework for managing global climate data^12^. Over 800+ individual datasets have been evaluated utilizing DSMM, including the WDS-Paleo International Tree-Ring Data Bank^11^.

### FAIR guiding principles

**Fig. 3:**
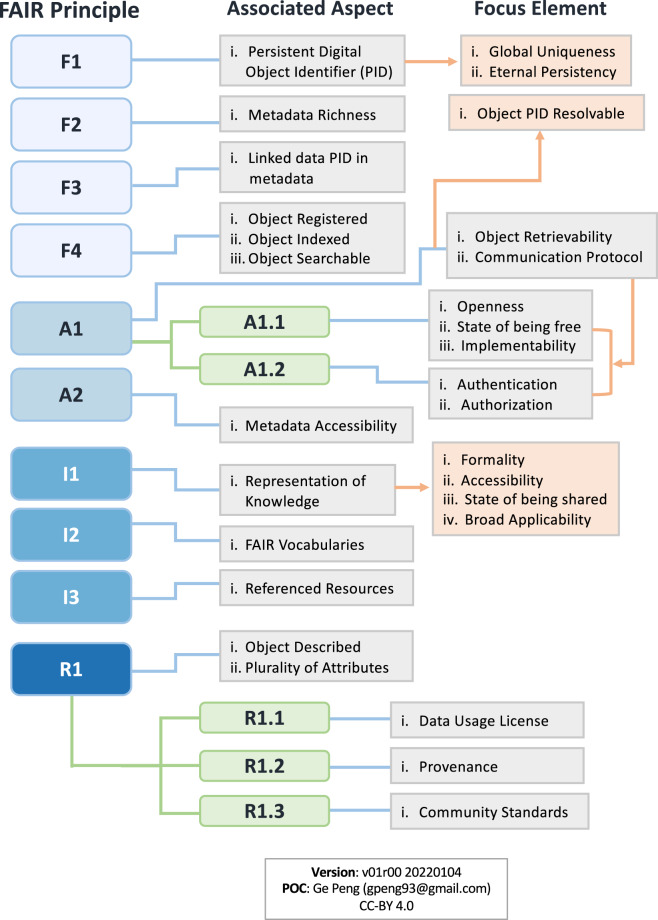
Diagram showing aspects (gray-filled text boxes) and elements (pink-filled text boxes) associated with each of the FAIR (i.e., Findable, Accessible, Interoperable, Reusable) Guiding Principles. The term “Object” in the diagram refers to “(meta)data”.

The FAIR Guiding Principles (‘FAIR Data Principles’ or simply ‘FAIR Principles’) were initiated at the 2014 Lorentz Workshop ‘Jointly Designing a Data FAIRport’ (https://www.openaire.eu/how-to-make-your-data-fair) and formally defined by stakeholders from academia, industry, funding agencies, and scholarly publishers to encourage data sharing across systems, disciplines, and regional boundaries^13^. They were developed after both CTDRR and DSMM. Like the latter, fifteen principles target individual datasets, but primarily concern individual practices or capabilities on quality attributes of findability, accessibility, interoperability, and reusability for enhanced data sharing in machine-friendly environments. These quality attributes are only a subset of what CTDRR and DSMM cover. The associated aspects and focus elements for each principle are shown in Fig. 3. The FAIR Principles have quickly gained popularity in the global data management and stewardship community, have been endorsed by many international organizations and countries, and have had a major impact in prompting data sharing and reuse worldwide^14–17^.

It should be noted here that while the FAIR Principles are not originally designed to be a stewardship maturity assessment approach per se, with many organizations already using the FAIR Principles in this way, they cannot be ignored from any comparison of predominant maturity models. Moreover, based on the FAIR Principles, an increasing number of tools and frameworks have been developed to assess the FAIRness of digital objects. For example, the Research Data Alliance (RDA) FAIR Data Maturity Model Working Group^18^ has developed a set of core assessment criteria for FAIRness. Known as RDA FAIR Data Maturity Indicators (DMIs), they aim to enable direct comparison of assessment results.

By examining the FAIR Principles at the highest level rather than one of the various tools derived from it, the expectation is to capture the major differences of all such approaches from CTDRR and DSMM, while limiting the complexity of resultant crosswalks.

### Disciplinary coverage of the approaches

Generally speaking, initial implementations of the FAIR Principles have tended to be focused on the Biological and Natural Sciences in Europe^19^, while implementations of the DSMM have mainly been for the Earth and Environmental Sciences^2^. CTDRR is completely agnostic when it comes to disciplinary coverage, data heterogeneity, and collection size. Its value as a general assessment approach has been proven by the highly diverse range of data repositories that have chosen to be certified using the CTDRR. A comprehensive cross-disciplinary suitability and relevance analysis of the three approaches could be a useful exercise, but it is beyond the scope of this paper.

## Results

A crosswalk analysis has been performed by comparing individual CTDRR Requirements, DSMM Key Components, and FAIR Principles with each criterion in turn of the other two approaches. In this section, an outline of the synergies in terms of strong or weak direct mappings among the three assessment approaches is provided along with a summary. A discussion of a strong versus weak mapping can be found in the Methods section.

### Synergies and distinctions between CTDRR and DSMM

While both were founded on the OAIS Reference Model, DSMM addresses stewardship at the dataset level, whereas CTDRR exemplifies an implementation of a TDR that then provides a tool for assessing the maturity of preservation and service capability at an organizational level. CTDRR thus helps set overarching stewardship processes, governance, and infrastructure for organizations that manage digital data objects over the long term^9^. Due to this difference in focus, knowledge of the similarities and distinctions between CTDRR and DSMM can enhance the ability of data stewards to develop synergistic strategies for the overall stewardship maturity of their repository and the data objects it encapsulates.

Synthesis was found by one of the authors between CTDRR and DSMM during the recertification process of NOAA/NCEI’s World Data Service for Paleoclimatology, with an observation that stewardship best practices captured in DSMM are beneficial in helping fulfill relevant requirements in CTDRR^20^. It is this observation that initiated the current research.

**Fig. 4:**
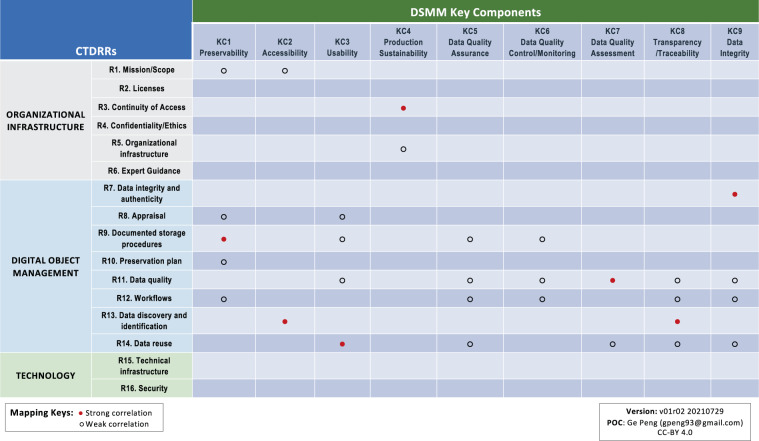
Crosswalk between CTDRR and DSMM. Strong (weak) correlations are denoted by filled (open) circles. Color-code under CTDRR: light gray/blue/green denotes the category of Organizational Infrastructure/Digital Object Management/Technology, respectively. Rn, n = 1, 2, …16, denote the CTDRR Requirements. KCn, n = 1, 2, …, 9 denote the DSMM Key Components.

As depicted in Fig. 4, the CTDRR Digital Object Management category (R7–R14) is the area that has the highest correlation to the DSMM KCs, with more than one pair of direct mappings between the two approaches. Because it is the category with the greatest concentration of Requirements that affect managing data objects—which is the main focus of DSMM on a level of individual datasets—six strong correlations are identified between the following CTDRR–DSMM pairs: (R7, KC9), (R9, KC1), (R11, KC7), (R13, KC2), (R13, KC8), and (R14, KC3). (More detailed reasonings are given in Supplementary Table 1).

By the same logic, since the CTDRR Requirements in the Technology category (R15–R16) concern software and hardware systems that provide services to a designated user community at the organizational level—which are outside the scope of DSMM—there are no direct correlations in this case. Likewise, there are only limited direct mappings between the CTDRR Requirements in the Organizational Infrastructure category (R1–R6) and the DSMM KCs, with only one strong correlation identified between R3 and KC4 (Fig. 4) because of the overlap between ensuring ongoing access to and preservation of the repository’s holdings via a continuity plan and a commitment to the data product being sustainable and extendable (Supplementary Table 1).

### Synergies and distinctions between CTDRR and FAIR Principles

**Fig. 5:**
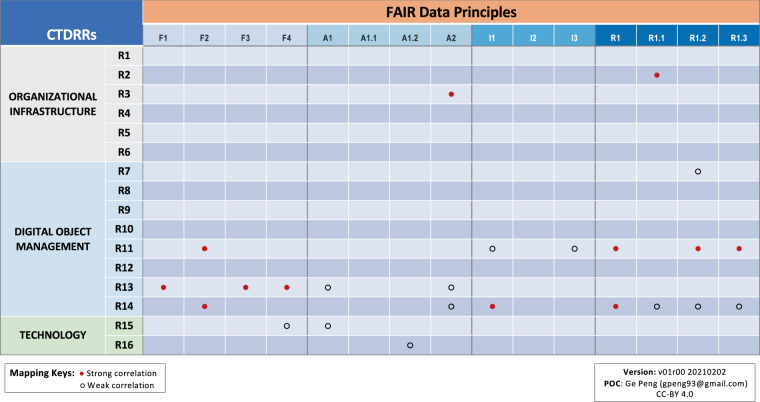
Crosswalk between CTDRR and FAIR Principles. Strong (weak) correlations are denoted by filled (open) circles. Color-code under CTDRR: light gray blue/green denotes the category of Organizational Infrastructure/Digital Object Management/Technology, respectively. Rn, n = 1, 2, …16, denote the CTDRR Requirements. {F, A, I, R}n[.m], denote the FAIR Principles.

It must be reiterated that these two approaches are concerned with different levels of data management. CTDRR is focused on a repository’s processes, capability, and infrastructure, whereas the FAIR Principles focus on the (collections of) digital objects themselves. Moreover, the ‘FAIRness’ of a digital object can be measured only at a specific moment. A TDR has the ability to maintain or even enhance the FAIRness of a digital object over time. In this regard, a repository may ‘enable’ FAIR. Because CTDRR takes a holistic organizational/ecosystem perspective, only small parts of it are picked up/touched on in the FAIR Principles (Fig. 5)—relationships exist in the sense that the FAIR Principles overlap with the set of best practices employed by TDRs.

Owing to the above, there is little crossover between the Organizational Infrastructure Requirements (R1–R6) of CTDRR and the FAIR Principles. In particular, the authors found no strong or weak correlations for R1 (Mission/scope), R4 (Confidentiality/ethics), R5 (Organizational infrastructure), and R6 (Expert guidance) of CTDRR (Fig. 5). The two strong correlations that were identified are between R2 of CTDRR and R1.1 of the FAIR Principles, and between CTDRR R3 and FAIR A2 (see also Supplementary Table 2).

The Digital Object Management Requirements (R7–R14) of CTDRR cover a repository’s practices for managing its digital objects. Consequently, the FAIR Principles are more directly relevant to these Requirements and there is greater overlap between the two approaches (Fig. 5). Specifically, 10 strong correlations are found between these CTDRR–FAIR Principles pairs: (R11, F2), (R11, R1), (R11, R1.2), (R11, R1.3), (R13, F1), (R13, F3), (R13, F4), (R14, F2), (R14, R1), and (R14, I1) (details are in Supplementary Table 2). There are some exceptions due to the more limited scope of the FAIR Principles, and the following CTDRR Requirements do not have analogous concepts: R8 (Appraisal), R9 (Documented storage procedures), R10 (Preservation plan), and R12 (Workflows).

Technology aspects are covered in Requirements R15 (Technical infrastructure) and R16 (Security) of CTDRR, and are enacted through organizational-wide protocols and procedures. Hence, they are at a repository’s infrastructural level and again are not really captured in the concept of FAIR. This means that there are no bi-directional, strong relationships between R15 and R16 and the FAIR Principles, but one can go rather weakly in a single direction in several cases.

### Synergies and distinctions between FAIR Principles and DSMM

**Fig. 6:**
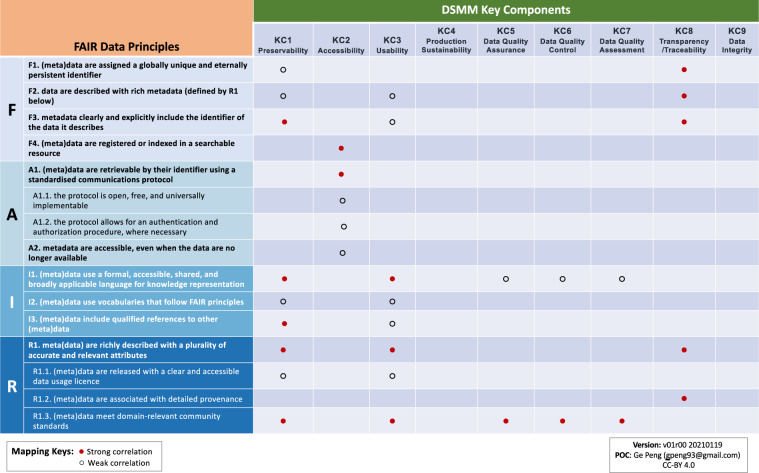
Crosswalk between FAIR and DSMM. Strong (weak) correlations are denoted by filled (open) circles. KCn, n = 1, 2, …, 9 denote the DSMM Key Components.

Strong synthesis was found between DSMM and the FAIR Principles, with stewardship practices captured in DSMM helping support the FAIRness of individual datasets (Fig. 6).

KC1 (Preservability) addresses aspects of long-term preservation, including data storage and archive practices and standards. KC3 (Usability) evaluates the level of data product-specific knowledge required to understand the data, their availability, and the comprehensiveness of documentation to support understanding in using them. KC8 (Transparency/Traceability) examines the state of being transparent, trackable, and traceable. Stewardship practices captured in KC1, KC3, and KC8 therefore help ensure (meta)data are assigned and referenced with unique persistent identifiers (PIDs; F1 and F3), are searchable online (F4), use broadly shared language for knowledge representation including provenance (R1.2) that conforms domain-relevant community or international standards (R1.3), while they are richly described (F2; R1) with a formal and shared language (I1) and referenced with other (meta)data (I3). All of this leads to there being strong correlations among one or more of these three KCs with three areas of the FAIR Principles; namely, F1–F3, I1, I3, R1, R1.2, and R1.3 (Fig. 6). (More detailed reasonings are given in Supplementary Table 3.)

KC2 (Accessibility) evaluates both the level of maturity in searchability and accessibility of data and associated data services. Stewardship practices captured in KC2 therefore strongly support FAIR data in ensuring the (meta)data are searchable (F4) and accessible with an open protocol (A1.1) that follows community standards (A1), and which allows for authentication and authorization (A1.2).

Practices defined in KC5, KC6, and KC7, respectively concerned with data quality assurance, control/monitoring, and assessment, further enhance the synthesis with FAIR in terms of utilizing formal knowledge representation (I1) that follows community standards (R1.3) (Fig. 6).

### Summary

A clear understanding of these three approaches for assessing stewardship maturity of digital data and repository processes is essential in (re)using them to cater to different data management and stewardship needs and end results.

CTDRR and DSMM are two of a handful of data stewardship maturity models used to ensure that best practices are followed for the acquisition, preservation, discovery, accessibility, interoperability, and reusability of data for the long-term. Both are based on the OAIS Reference Model. They mostly overlap in the area of managing digital data objects, providing conditions for various levels of maturity that can be utilized as metrics to enhance the management processes for a repository’s data holdings. The main differences between these two approaches stem from their different perspectives.

In contrast, the FAIR Principles provide high-level guidance for enabling that (meta)data are findable, accessible, interoperable, and reusable, and prompting data sharing in a machine-friendly environment. They have the most limited scope of the three approaches, and tend to measure the end state in a binary fashion; specifically, whether a Principle is satisfied or not. Unlike CTDRR and DSMM, they do not include maturity levels for measurement of individual FAIR Principles nor do they evaluate data quality practices explicitly. Nonetheless, as shown in the Introduction, the Principles are extremely important in fostering data sharing and reuse.

Strong correlations among these three assessment approaches are found in terms of data discovery, accessibility, interoperability, and usability as a result of their overlapping requirements for the stewardship of data objects.

From an institutional perspective, a prime objective of TDRs is to meet certification requirements—such as CTDRR—as well as codify organizational governance and management plans for stewarding data for the long term. In tandem with the data object perspective, creation and stewardship lifecycle management best practices can be guided by all three stewardship approaches, increasing in granularity from DSMM and FAIR to CTDRR.

## Discussion

### One-directional correlations

Bi-lateral correlations are found for most strong correlations but do not always hold. For example, the strong correlations from DSMM KC5–7 to FAIR R1.3 (Fig. 6) are the result of practices captured in DSMM for conforming to community quality metadata standards. However, meeting domain *metadata* standards^21^ does not necessarily or automatically lead to conforming to domain *quality metadata* standards^22^.

### Inferred correlations

**Fig. 7:**
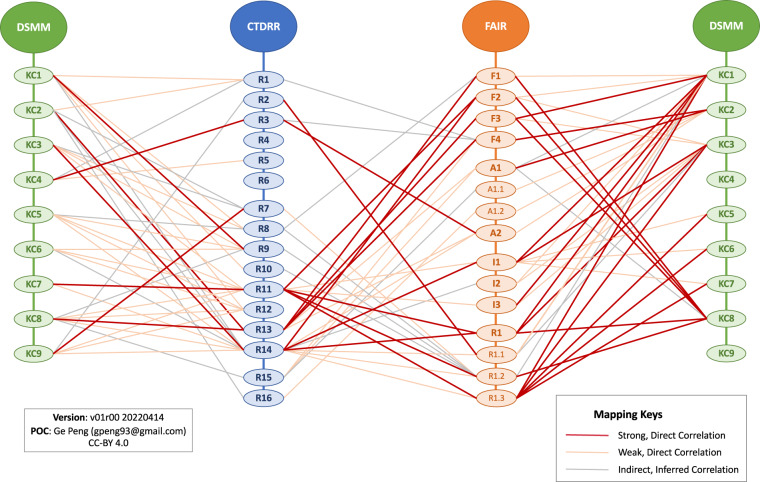
Mappings between each pair of CTDRR (Requirements denoted by blue ovals), FAIR (Principles by orange ovals), and DSMM (Key Components by green ovals).

Extra to the strong and weak direct correlations among the approaches, the authors found a number of indirect correlations due to subjective interpretations within the assessment approaches. Such inferences lead to complexity in analyzing individual approaches and to many potential crosswalks among them according to different efforts. Shown as an example, Fig. 7 depicts the inferred crosswalks among CTDRR, DSMM, and FAIR from our exercise (thin gray lines) in addition to the strong (thick red lines) and weak (thin pink lines) direct mappings—the majority are highly subjective, and interpretation of individual criteria diverged even among the authors.

A specific instance of an inferred relationship can be seen with DSMM KC1 (Preservability), which contains verbiage of being compliant to community archival metadata standards. Such metadata standards can require a description of data lineage equivalent to provenance (FAIR R1.2), but whether this leads to a direct mapping with FAIR I3 (including cross-references) is subtle due to the assumption of what the underlying standards entail. Similarly, DSMM KC3 (Usability) touches on the need for a standard-based interoperable data format and metadata, which can potentially contain a usage license and thus map to FAIR R1.1. Another inferred mapping might be found between CTDRR R14 (Data Reuse) and FAIR I2, since appropriate metadata to support understanding and (re)use of data holdings (R14) may partially and implicitly explain the need for the existence of controlled vocabularies.

### Scope of the chosen approaches

It is worth noting that both CTDRR and DSMM go beyond just enabling FAIR. For example, DSMM KC5–7 aim to ensure data product quality by defining and implementing quality assurance, control, or assessment procedures, and promote transparency by emphasizing the need to document these procedures. Furthermore, two areas of DSMM are not explicitly covered by the FAIR Principles—the funding and commitment for sustainability of data products (KC4) and requirements for data fixation and authenticity verification (KC9)—although the latter may be inferred from R1.2 (detailed provenance) and A1.2 (the communication protocol allows for an authentication and authorization procedure). In the case of CTDRR, even within the digital object management category, there are no direct mappings from R8 (Appraisal), R9 (Documented Storage Procedures), and R10 (Preservation Plan) to any of the FAIR Principles, despite these requirements being critical to the long-term preservation of digital objects.

## Methods

A crosswalk analysis was performed, comparing individual CTDRR Requirements, DSMM Key Components, and FAIR Principles with each criterion in turn of the other two approaches. The goal was to identify similar data stewardship practices among these three maturity assessment approaches, with direct correlations between criteria categorized as strong or weak/some. The three authors have significant expertise and practical experience in the area of data stewardship maturity assessment, with in-depth knowledge of all three approaches. In all cases, the authors were required to reach consensus on each correlation by using the explicit definitions contained in CTDRR, DSMM, and the FAIR Principles as high-level criteria for subjectively determining direct crosswalks on focus areas or elements. Potential implementations or interpretations of each requirement/principle/key component were not explored as they would have drastically increased the complexity of resultant crosswalks.

The reasonings for each direct crosswalk between CTDRR and DSMM, where they exist, are captured in Supplementary Table 1; between CTDRR and FAIR are captured in Supplementary Table 2; and between FAIR and DSMM are captured in Supplementary Table 3. Strong correlation was identified when there was significant overlap between the two conditions being compared. Weak or some correlation was identified when a correlation was present but was only partially overlapping or held in specific instances. Through these methods, we drew out the general conclusions of crosswalks among the three approaches shown in Figs. 4–6 and described in the Results section.

## Supplementary information

Supplementary Table 1

Supplementary Table 2

Supplementary Table 3

## Data Availability

No data was generated.

## Code availability

No custom code was generated.

## References

[1] Peng, G., Privette, J. L., Kearns, E. J., Ritchey, N. A. & Ansari, S. A unified framework for measuring stewardship practices applied to digital environmental datasets. *Data Sci J* **13**, (2015).

[2] Peng, G. The state of assessing data stewardship maturity – An overview. *Data Sci J* **17** (2018).

[3] Peng, G. *et al*. Call to action for global access to and harmonization of quality information of individual earth science datasets. *Data Sci J* **20** (2021).

[4] ISO 16360. *Space data and information transfer systems–Audit and certification of trustworthy digital repositories*, https://www.iso.org/standard/56510.html (2012).

[5] CCSDS. *Reference Model for an Open Archival Information System (OAIS), Recommended Practices*, *Issue 2*., https://public.ccsds.org/pubs/650x0m2.pdf (2012).

[6] Edmunds R, L’Hours H, Rickards L, Trilsbeek P, Vardigan M (2016). Zenodo.

[7] CoreTrustSeal. *Core Trustworthy Data Repository Requirements 2017–2019 - Extended Guidance*. (2017).

[8] CoreTrustSeal (2019). Zenodo.

[9] Lin, D. *et al*. The TRUST Principles for digital repositories. *Sci Data* **7**, 144 (2020). 10.1038/s41597-020-0486-7 PMC7224370 32409645

[10] WMO SMM-CD Working Group (2019). Figshare.

[11] Peng, G. *et al*. Practical application of a data stewardship maturity matrix for the NOAA OneStop project. *Data Sci J* **18** (2019).

[12] WMO. *Manual on the High-Quality Global Data Management Framework for Climate. Document ID: WMO-No.1238*., https://library.wmo.int/doc_num.php?explnum_id=10197 (2019).

[13] Wilkinson, M. D. *et al*. The FAIR Guiding Principles for scientific data management and stewardship. *Sci Data* **3**, 160018 (2016). 10.1038/sdata.2016.18 PMC4792175 26978244

[14] G20 Leaders. *G20 Leaders’ Communique Hangzhou Summit*., https://ec.europa.eu/commission/presscorner/detail/en/STATEMENT_16_2967 (2016).

[15] Australia FAIR Access Working Group. *Policy Statement on FAIR Access to Australia’s Research Outputs*., https://www.fair-access.net.au/fair-statement (2017).

[16] European Commission. *Turning FAIR into reality - Final Report and Action Plan from the European Commission Expert Group on FAIR data*., 10.2777/1524 (2018).

[17] Mons, B. *Data Stewardship for open science: implementing FAIR principles*. (Chapman and Hall/CRC Press, Taylor & Francis, 2018).

[18] RDA FAIR Data Maturity Model Working Group. *FAIR Data Maturity Model: specification and guidelines*., 10.15497/rda00045 (2014).

[19] van Reisen M (2020). Towards the tipping point for fair implementation. Data Intell.

[20] NCEI. *World Data Service for Paleoclimatology - CoreTrustSeal certification evaluation*., https://www.coretrustseal.org/wp-content/uploads/2020/02/World-Data-Service-for-Paleoclimatology.pdf (2020).

[21] ISO 19115-1. *Geographic Information—Metadata - Part 1: Fundamentals*., https://www.iso.org/standard/53798.html (2014).

[22] ISO 19157. *Geographic information - Data quality*., https://www.iso.org/standard/32575.html (2013).

